# Dopamine modulates individual differences in avoidance behavior: A pharmacological, immunohistochemical, neurochemical and volumetric investigation

**DOI:** 10.1016/j.ynstr.2020.100219

**Published:** 2020-04-08

**Authors:** Geiza Fernanda Antunes, Flavia Venetucci Gouveia, Fabiana Strambio Rezende, Midiã Dias de Jesus Seno, Milene Cristina de Carvalho, Caroline Cruz de Oliveira, Lennon Cardoso Tosati dos Santos, Marina Correia de Castro, Mayra Akemi Kuroki, Manoel Jacobsen Teixeira, José Pinhata Otoch, Marcus Lira Brandao, Erich Talamoni Fonoff, Raquel Chacon Ruiz Martinez

**Affiliations:** a Division of Neuroscience, Hospital Sirio-Libanes, Sao Paulo, Brazil; b University of Sao Paulo and Institute of Neuroscience and Behavior (INeC), Campus USP, Ribeirao Preto, Brazil; c Department of Neurology, Division of Functional Neurosurgery, Institute of Psychiatry, University of Sao Paulo School of Medicine, Sao Paulo, Brazil; d Department of Surgery Techniques, School of Medicine, University of Sao Paulo, Sao Paulo, Brazil; e LIM/23 – Laboratório de Psicopatologia e Terapêutica Psiquiátrica, Institute of Psychiatry, University of Sao Paulo School of Medicine, Sao Paulo, Brazil; f UNICID – Universidade Cidade de São Paulo, Sao Paulo, Brazil

**Keywords:** Anxiety disorders, Avoidance, Amygdala, Prefrontal cortex, Dopamine, Aversive learning

## Abstract

Avoidance behavior is a hallmark in pathological anxiety disorders and results in impairment of daily activities. Individual differences in avoidance responses are critical in determining vulnerability or resistance to anxiety disorders. Dopaminergic activation is implicated in the processing of avoidance responses; however, the mechanisms underlying these responses are unknown. In this sense, we used a preclinical model of avoidance behavior to investigate the possibility of an intrinsic differential dopaminergic pattern between good and poor performers. The specific goal was to assess the participation of dopamine (DA) through pharmacological manipulation, and we further evaluated the effects of systemic injections of the dopaminergic receptor type 1 (D1 antagonist - SCH23390) and dopaminergic receptor type 2 (D2 antagonist - sulpiride) antagonists in the good performers. Additionally, we evaluated the effects of intra-amygdala microinjection of a D1 antagonist (SCH23390) and a D2 antagonist (sulpiride) in good performers as well as intra-amygdala microinjection of a D1 agonist (SKF38393) and D2 agonist (quinpirole) in poor performers. Furthermore, we quantified the contents of dopamine and metabolites (3,4-dihydroxyphenylacetic acid (DOPAC) and homovanillic acid (HVA)) in the amygdala, evaluated the basal levels of tyrosine hydroxylase expression (catecholamine synthesis enzyme) and measured the volume of the substantia nigra, ventral tegmental area and locus coeruleus. Our results showed that it could be possible to convert animals from good to poor performers, and vice versa, by intra-amygdala (basolateral and central nucleus) injections of D1 receptor antagonists in good performers or D2 receptor agonists in poor performers. Additionally, the good performers had lower levels of DOPAC and HVA in the amygdala, an increase in the total volume of the amygdala (AMG), substantia nigra (SN), ventral tegmental area (VTA) and locus coeruleus (LC), and an increase in the number of tyrosine hydroxylase-positive cells in SN, VTA and LC, which positively correlates with the avoidance behavior. Taken together, our data show evidence for a dopaminergic signature of avoidance performers, emphasizing the role of distinct dopaminergic receptors in individual differences in avoidance behavior based on pharmacological, immunohistochemical, neurochemical and volumetric analyses. Our findings provide a better understanding of the role of the dopaminergic system in the execution of avoidance behavior.

## Highlights

• The role of dopamine in individual differences in avoidance behavior.
• Dopamine modulates avoidance behavior.
• Dopaminergic evidence of individual difference in avoidance behavior.
• Good and poor avoiders distinction based on dopaminergic signature.
• Dopaminergic signature of avoidance performers: poor versus good avoiders.

## 1 Introduction

Avoidance behavior consists of the transition from fear reactions to motor actions to avoid a harmful or unpleasant stimulus, increasing the animal's chance of survival in the face of potential damage (Skinner, 1969; Lang et al., 1998). It has been observed in different species, including humans, primates and rodents (LeDoux, 1996; Sheynin et al., 2014). Conversely, the manifestation of avoidance responses in the absence of real potential danger is the central characteristic of several mental disorders (e.g., panic disorder, avoidant personality disorder) (World Health Organization, 2004; Lampe, 2016) and is related to anxiety disorders (e.g., social anxiety disorder and social phobia) (Moitra et al., 2008; American Psychiatric Association, 2013; Bardeen et al., 2014).

Preclinical models of avoidance behavior are fundamental to better understand the course and etiology of the behavior and to provide insights for new human pharmacological treatments (Krypotos et al., 2015). The two-way active avoidance task is performed in a shuttle box that is divided into two compartments by a door, and the rats are trained to exhibit the avoidance behavior by moving from one compartment to another in order to avoid the delivery of the footshocks (Martinez et al., 2013). This is a particularly interesting model because two different subpopulations are distinguished based on the avoidance response, the good (high avoiders) and poor (low avoiders) performers, with higher levels of anxiety displayed by the poor performers (Martinez et al., 2013). Therefore, in the context of post-traumatic stress disorder (PTSD) and other anxiety disorders, the distinction between good and poor performers could be helpful to study the persistent and maladaptive threat responses (Mahan and Ressler, 2012; Galatzer-Levy et al., 2014).

It is suggested that a fronto-limbic-striatal network controls this behavior and that maladaptive responses could be due to a reduction in frontal and limbic activity, especially related to the amygdala and prefrontal cortex (Schlund et al., 2011; Martinez et al., 2013; Ramirez et al., 2015). The central (Ce), basolateral (BLA) and basomedial (BMA) amygdala nuclei are critical for the modulation of avoidance behavior (Choi et al., 2010; Martinez et al., 2013; Ramirez et al., 2015; Jiao et al., 2015). In addition, the close involvement of the ventral tegmental area, nucleus accumbens and habenula to this neurocircuitry points to a possible dopaminergic modulation of avoidance behavior (Carlezon and Thomas, 2009; Darvas et al., 2011; Stamatakis and Stuber, 2012; Fernando et al., 2014; Sanna et al., 2014; 2017). In fact, dopamine (DA)-deficient mice have impaired avoidance behavior, and the restoration of the DA system in the amygdala and striatum is sufficient to restore this behavior (Darvas et al., 2011). In this sense, dopaminergic activation in the ventral tegmental area and substantial nigra has been implicated in processing avoidance response (Rigoli et al., 2016); however, the mechanisms underlying these responses are unknown.

Considering the key role of avoidance in normal and psychiatric status, it is critical to improve our understanding of the mechanisms responsible for modulating avoidance response. To investigate the participation of DA through pharmacological manipulation, we evaluated the effects of systemic injections of a D1 antagonist (SCH23390) and a D2 antagonist (sulpiride) in the good performers. Additionally, we evaluated the effects of intra-amygdala microinjection of dopaminergic receptor type 1 (D1) antagonist (SCH23390) and a dopaminergic receptor type 2 (D2) antagonist (sulpiride) in the good performers and intra-amygdala microinjection of a D1 agonist (SKF38393) and a D2 agonist (quinpirole) in poor performers. Furthermore, to assess the possibility of an intrinsic differential DA pattern between good and poor performers, we quantified DA and its metabolites (3,4-dihydroxyphenylacetic acid (DOPAC) and homovanillic acid (HVA) in the amygdala, evaluated the basal levels of tyrosine hydroxylase (TH) expression (catecholamine synthesis enzyme) and measured the volumes of the substantia nigra, ventral tegmental area and locus coeruleus.

## 2 Materials and methods

### 2.1 Subjects

Male Wistar rats (N = 246) from the animal facility of the University of Sao Paulo were used as subjects. The animals weighed 200–300 g and were housed in polypropylene cages (40 × 34 × 17 cm) in groups of three under a 12:12 dark/light cycle (lights on at 07:00 h), room temperature maintained at 24 °C ± 1 °C, wood shavings and free access to food and water throughout the experiment. Animals were maintained in the animal facility for 7 days before experiments for habituation. The experiments reported in this paper were performed in compliance with the recommendations of the Brazilian Society of Neuroscience and Behavior, which, in turn, are based on the US National Institutes of Health Guide for Care and Use of Laboratory Animals. The study was approved by the Ethics Committee on the Use of Animals at Hospital Sirio-Libanes (CEUA #2013/12) and the Medical School of the University of Sao Paulo (CEP #083/11).

### 2.2 Apparatus/procedure

#### 2.2.1 Sidman active avoidance

Behavioral training/testing was conducted in two-way shuttle boxes (Insight Equipments, Brazil). Animals were randomly assigned for experimental or box control; experimental animals were submitted to seven 25-min daily training sessions in the two-way shuttle box and tested on the eighth day; control animals received equivalent exposure to the box without the footshock delivered. Shuttling between compartments delayed the delivery of scrambled footshock unconditioned stimulus - US (0.6 mA; 0.5 s) by 30 s (R–S interval). In the absence of shuttling, US delivery occurred every 5 s. The response to stimulus (R–S) interval shuttles comprised avoidance responses and stimulus to stimulus (S–S) interval shuttles comprised escape responses. All shuttles produced 0.3 s feedback stimuli (house light blink). Animals were divided into good and poor performance groups according to the number of avoidance responses exhibited in the last two training sessions. The animals that performed more than 20 avoidance responses were considered good performers, while animals that did not achieve this number were considered poor performers (Lázaro-Muñoz et al., 2010; Martinez et al., 2013; de Oliveira et al., 2016). Freezing was defined as the absence of movement except that required for breathing (Blanchard and Blanchard, 1969) and it was assessed during the first 2 min of the tests based on a previous study (Lázaro-Muñoz et al., 2010). The evaluation was performed by an observer blinded to the group specification.

#### 2.2.2 Open field test

After the test at day 8 on the two-way shuttle boxes, animals were evaluated in the open field for general motor activity. The apparatus consists of a 0.6 m square dark gray Formica surrounded by 50-cm high Formica walls. Each rat was placed in the center of the open field and allowed to explore it freely for 10 min. Sessions were recorded with a video camera for future analysis. After each animal completed the test, the open field was cleaned with 5% ethanol and then dried with a dry cloth.

#### 2.2.3 Dopaminergic drugs

For the pharmacological manipulation of avoidance behavior, the following drugs were used: (see Table 1)

**Table 1:** Drugs and doses used for pharmacological DA manipulation.

Type of Drug	Intraperitoneal Injection (Name/Dose)	Intra-amygdala Injection (Name/Dose)
D1 agonist	NA	(SKF38393, Tocris) 0.4 μL
D1 antagonist	(SCH23390, Sigma-Aldrich) 0.025 mg/kg and 0.05 mg/kg	(SCH23390, Sigma-Aldrich) 0.3 μL (0.1 μg/0.1 μL)
D2 agonist	NA	(Quinpirole, Tocris) 0.1 μL
D2 antagonist	(Sulpiride, Sigma-Aldrich) 20 mg/kg and 40 mg/kg	(Sulpiride, Tocris) 0.2 μL
Saline	Sterile Saline Solution (0.9%) 10 mL/kg	Sterile Saline Solution (0.9%) 0.1–0.4 μL

Abbreviations: DA: dopaminergic; NA: not applicable; D1: dopamine receptor 1; D2: dopamine receptor 2. Intra-amygdala drug dilution 1:1.

#### 2.2.4 Cannula implantation

Animals were anesthetized with isoflurane (4% induction; 2% maintenance in 100% oxygen) through a face-mask attached to the stereotaxic instrument (David Kopf, Germany). For optimizing analgesia, immediately after hair trichotomy, animals had received local anesthetic (0.1 mL of Lidocaine chlorhydrate with norepinephrine, Cristalia, Brazil) and were injected intraperitoneally with 0.1 mL of morphine sulfate (10 mg/mL, Cristalia, Brazil). The animal's scalp was cleaned with 10% iodopovidone, the skull was exposed and the periosteum was removed. Using a dental drill (LB-100; Beltec - Brazil), bilateral holes were made in the skull for the implantation of the cannula directed to the basolateral (AP: 3.0 mm; ML: 5.1 mm; DV: 7.0 mm) or central (AP: 2.4 mm; ML: 4.0 mm; DV: 7.0 mm) nuclei of the amygdala, following the coordinates described at Paxinos and Watson Atlas (Paxinos and Watson, 2005). The cannulas were fixed with acrylic resin (JET, Brazil) and two screws previously fixed and sealed with a stainless steel wire to protect it against obstruction, which was removed at the time of the microinjection.

After surgery, animals were injected subcutaneously with 0.3 mL broad-spectrum veterinarian Pentabiotic Reinforced (Wyeth, Brazil) and received sodium dipyrone (50 mg/mL, Medley) diluted in sterile injection water (final concentration: 2.5 mg/mL) for three consecutive days. Animals were allowed to recover from surgery for seven days.

#### 2.2.5 Amygdala drugs microinjection

On the test day (8th day), the cannula seal was removed from the animal's head, and an infusion needle was introduced (1 mm below the guide cannula) for solution injection. An infusion pump (Harvard Apparatus) with a flow rate of 0.25 μL/min was used for drugs and saline microinjection. The infusion needle was kept in place for one extra minute after the end of the microinjection to maximize the diffusion process (Macedo et al., 2005). After a 10-min interval, animals were tested on the two-way shuttle box for avoidance and freezing behavior evaluation. Poor performers received microinjections of DA agonists (quinpirole; SKF38393) or saline in the basolateral or central nuclei of the amygdala, while good performers received DA antagonist microinjections (SCH23390; sulpiride) or saline in the same targets.

#### 2.2.6 Systemic drug administration

On the test day (8th day), good performers were intraperitoneally injected with dopaminergic receptor antagonist (SCH23390 30-min interval; sulpiride 10-min interval) or saline and tested in the two-way shuttle box for avoidance and freezing behaviors evaluation (de Souza Caetano et al., 2013).

#### 2.2.7 Brain extraction

##### 2.2.7.1 Perfusion

Ninety minutes after testing, the animals were deeply anesthetized with thiopental (40 mg/kg) and morphine sulfate (10 mg/mL) and perfused transcardially with a solution of 4.0% paraformaldehyde in 0.1 M phosphate buffer, using a peristaltic pump (Cole Parmer). Brains were removed, placed in paraformaldehyde for 3 h, and then transferred to a 30% sucrose/0.1 M phosphate buffer at 4 °C. Frozen whole brain coronal sections (40 mm thick) were sliced with a sliding microtome (Leica Biosystems).

##### 2.2.7.2 Decapitation

Animals were deeply anesthetized with thiopental (40 mg/kg) and morphine sulfate (10 mg/mL), and heads were removed using a rodent guillotine (Insight Equipments, Brazil). After removal from the skull, brains were placed on an iced brain matrix and cut (1 mm), according to bregma reference (Paxinos and Watson, 2005). Samples were weighed and kept in a deep freezer (−80 °C) until analysis.

#### 2.2.8 C-fos and TH immunohistochemistry

Brain sections were processed with anti-c-Fos antiserum raised in rabbit (Ab-5, Calbiochem, lot-D07099; dilution 1:20,000) or anti-TH raised in mouse (Millipore; Lot-LV1679333; dilution 1:1000). Primary antiserum was localized using an avidin–biotin complex system (ABC; Vector Laboratories). Briefly, sections were incubated in biotinylated goat anti-rabbit IgG solution (Vector Laboratories), then placed in the mixed avidin–biotin horseradish peroxidase complex solution (ABC Elite Kit; Vector Laboratories) (90 min/step at 22 °C). The peroxidase complex was incubated in a chromogen solution containing 0.02% 3,3′-diaminobenzidine tetrahydrochloride (DAB; Sigma) with 0.3% nickel-ammonium sulfate in 0.05 M Tris-buffer (pH 7.6), followed by incubation in this chromogen solution with hydrogen peroxide (1:3000) (10 min/step) (Introini-Collison et al., 1979). Extensive washing in PBS buffer (pH 7.4) halted the DAB reaction. Sections were mounted on gelatin-coated slides, dehydrated, and coverslipped with DPX (Sigma). An adjacent reference series was Nissl stained for cytoarchitectonic purposes.

#### 2.2.9 Quantification

For c-Fos-immunoreactive neuron quantification, brain slides were scanned using Pannoramic and Case Viewer (Biogen), and images were processed using ImageJ (News Version 1.44b). c-Fos positive neuron density was determined by dividing the resulting number by the area of the region of interest (delimited based on the Paxinos and Watson atlas) (Paxinos and Watson, 2005). The structures evaluated along the anteroposterior axis were prefrontal cortex (prelimbic and infralimbic) in bregma: 3.00 mm, 3.75 mm and 3.72 mm and amygdaloid nuclei (basolateral, lateral, medial and central) in bregma: 2.04 mm, −2.28 mm, −2.76 mm and −3.00 mm. For the TH quantification, photomicrographs of brain slides were taken using a 10x objective of a microscope equipped with Camera Lucida (NIKON ECLIPSE E600). The count was performed using ImageJ (News Version 1.44b) following the same protocol described above. The structures evaluated along the anteroposterior axis were the SN, VTA and LC in bregma: 6.72 mm; −10.08 mm; −9.96 mm. All the quantifications were performed by an observer blinded to the behavioral results.

#### 2.2.10 High-performance liquid chromatography (HPLC)

The HPLC system was equipped with a reversed-phase column (Hypurity Elite C18, 250 mm × 4.6 mm, 5 μm and 100 Å pore-diameter particle size; Hypersil, Cheshire, United Kingdom) together with electrochemical detection. The samples were homogenized in 0.2 M perchloric acid containing dihydroxybenzylamine (DHBA) and centrifuged at 15,000 rpm for 20 min at 6 °C. A 50 μL sample dilution was injected into the HPLC system along with a proper buffer (150 mM chloroacetic acid, 120 mM NaOH, 0.67 mM EDTA, 0.86 mM sodium octylsulfate, 3.5% acetonitrile, 2.6% tetrahydrofuran; adjusted to pH 3.0) at a flow rate of 1.2 mL/min. The maximum sensitivity of the electrochemical detector was set at 2 nA, and the electrode oxidation potential was set at 850 mV versus a reference electrode. The ratios of DOPAC/DA and HVA/DA were used as indices of dopaminergic activity (Carvalho et al., 2005).

#### 2.2.11 Volumetric analysis

Brain sections were mounted on gelatin-coated slides and Nissl stained according to the protocol previously described (Paul et al., 2008). Corresponding bregma slices were selected for evaluation, based on The Rat Brain Atlas (Paxinos and Watson, 2005), as follows: 1. Amygdala: bregma −1.20 to −4.08; 2. SN: bregma −4.36 to −6.72; 3. VTA: bregma −4.68 to −6.84; 4. LC: bregma −9.48 to −10.32. Images were acquired using a Digital Microscope Zeiss AxioCam Plus (exposure: 28 ms; length: 2.5 × 1300 zvs) and analyzed with the software AxioVision 3.0.6.1©1998–2000 (Carl Zeiss Vision Microimaging GmbH, Germany). The final volume estimation was determined as the sum of all bregma measures for that specific structure, considering the interval between slices (150 μm), as described in a previous work (Keeley et al., 2015).

#### 2.2.12 Statistical analyses

Data are presented as the mean ± standard error of the mean (SEM). Freezing time, number of avoidance responses and administration of drugs were analyzed with two-way repeated measure analysis of variance ANOVA (factors: Group and Session). Data obtained in the open field, dopamine and metabolites quantification, volume and TH staining and c-Fos pattern were evaluated with the one-way ANOVA test. Correlations were set using the Pearson correlation test. When necessary, the Newman-Keuls post hoc test was applied. Significance was set at p < 0.05.

### 2.3 Study design

**Fig. 1:**
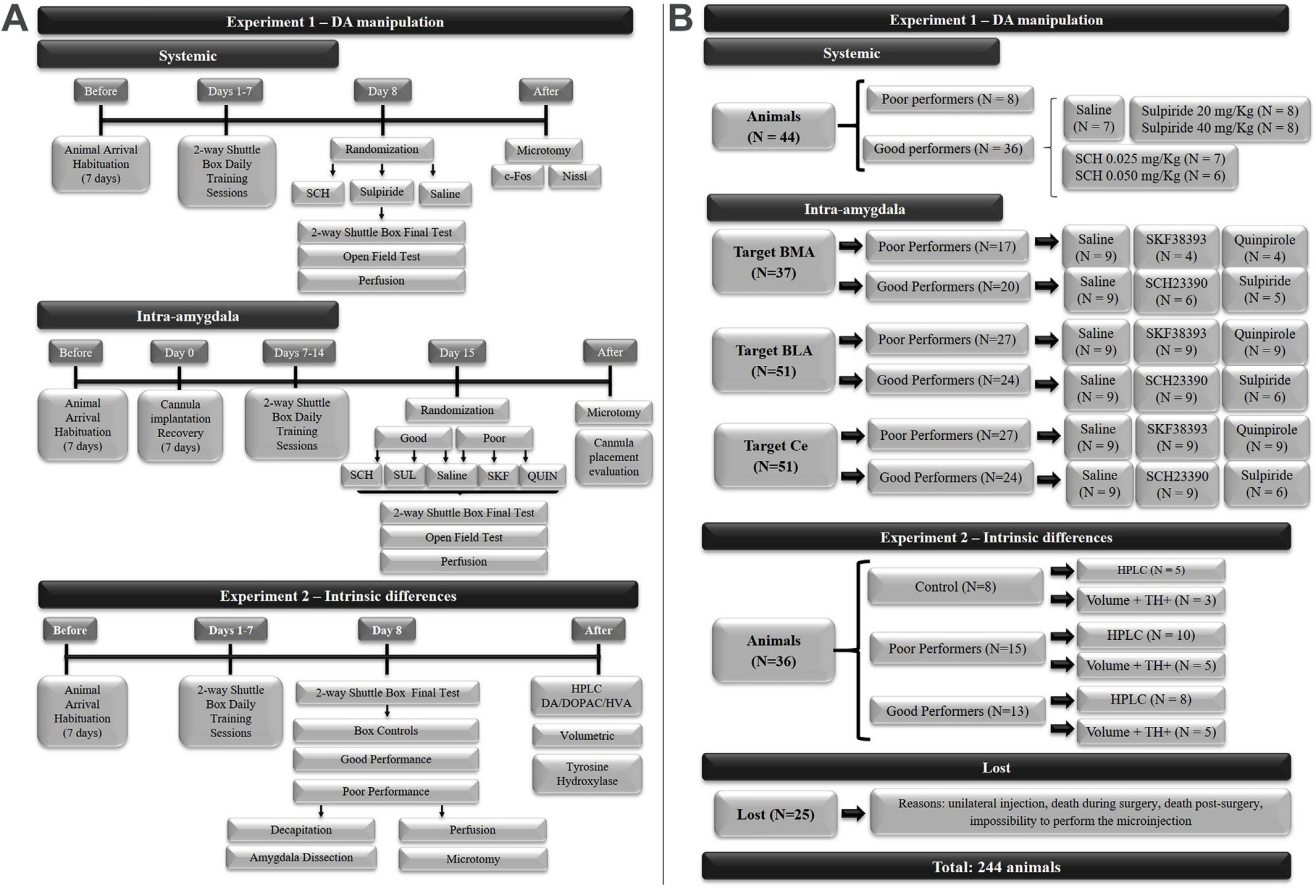
(A). Experimental design of the study divided in Experiment 1 that consisted of dopaminergic manipulation that was performed systemic and intra-amygdala manipulation in good and poor performers; and Experiment 2 that evaluated intrinsic differences. (B). Flowchart of the animals used in the study illustrating the number of the animals used per experiment in each experimental condition. QUIN: quinpirole. SCH: SCH23390; SKF: SKF38393.

The study design is shown in Fig. 1A.

### 2.4 Experiment 1 – dopaminergic manipulation

#### 2.4.1 Systemic pharmacological DA manipulation

As shown in Fig. 1A, after the habituation period in our animal facility, animals were submitted to seven daily training sessions in the two-way shuttle box and subsequently divided in good and poor performance according to the behavioral response exhibited. On the eighth day, good performers were randomly assigned to the control or experimental group and systemic administration of saline or dopaminergic antagonists, respectively. After a standard period of time, animals were evaluated in the two-way shuttle box and in the open field test. Ninety minutes after testing, animals were perfused; brains were removed, sliced in a freezing microtome and processed for c-Fos immunohistochemistry and Nissl staining.

#### 2.4.2 Intra-amygdala pharmacological DA manipulation

After the habituation period in our animal facility, animals were submitted to a surgical procedure for the implantation of the infusion cannula. Consecutively to the surgical recovery period, animals were submitted to seven daily training sessions in the two-way shuttle box and divided in good and poor performance according to the number of avoidance responses exhibited in the last two sessions. On the eighth day, animals were randomly assigned to the control or experimental group and administered saline or pharmacological agents. Good performers assigned for the experimental group received dopaminergic antagonists and poor performers assigned for the experimental group received dopaminergic agonists. After a standard period of time, animals were evaluated in the two-way shuttle box and in the open field test. Immediately after testing, animals were perfused; brains were removed, sliced in freezing microtome and processed for Nissl staining for validation of cannula placement.

### 2.5 Experiment 2 –basal DA levels

As shown in Fig. 1A, after a habituation period in the animal facility, the animals were randomly divided into experimental or control groups. Animals in the experimental group were submitted to eight daily training sessions in the two-way shuttle box and subsequently divided in good and poor performance groups according to the behavioral response exhibited in the last two sessions. Control animals were equally exposed to the shuttle box but never received any footshock.

On the last day, immediately after testing, a portion of the animals was decapitated, the brain was removed, and the amygdala was dissected for the quantification of dopamine and dopamine metabolites by HPLC. Another portion of the animals was perfused; the brains were removed, sliced in a freezing microtome and processed for TH immunohistochemistry and volumetric analysis.

## 3 Results

The number of animals used in each Experiment is shown in Fig. 1B.

### 3.1 Experiment 1 – DA manipulation

#### 3.1.1 Behavior assessment

**Fig. 2:**
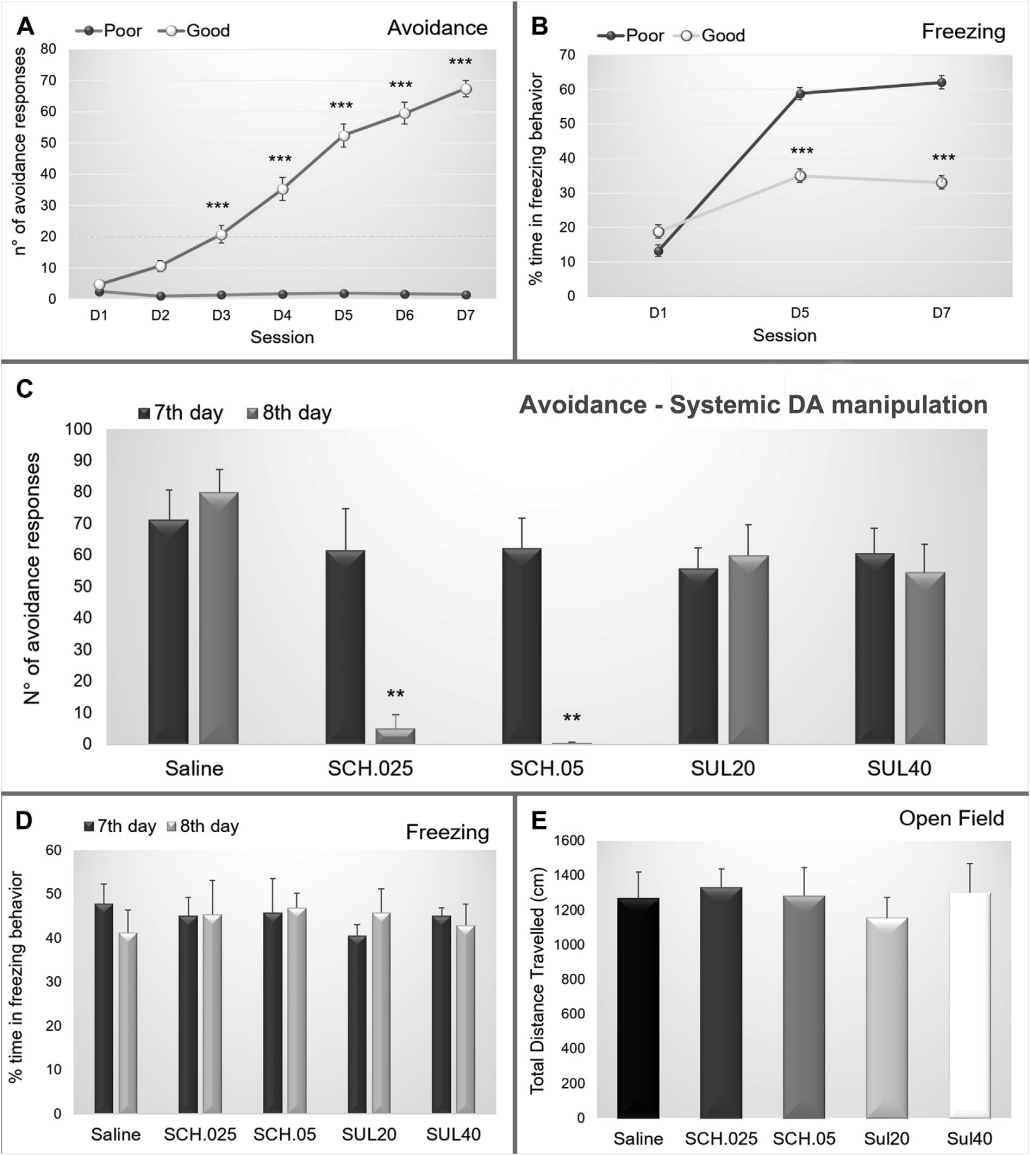
(A) Number of avoidance and (B) Percentage of the time spent in freezing exhibited by the poor and good avoiders. (C) Number of avoidance performed and (D) Percentage of time spent in freezing behavior by the good performers on the 7th day of training and on the 8th day of training after the administration of saline (N = 7), SCH 0.025 mg/kg (N = 7), SCH 0.05 mg/kg (N = 6), sulpiride 20 mg/kg (N = 8) or sulpiride 40 mg/kg (N = 8). (E) Total travel distance showed by good performers after the administration saline (N = 7), SCH 0.25 mg/kg (N = 7), SCH 0.5 mg/kg (N = 6), sulpiride 20 mg/kg (N = 8) or sulpiride 40 mg/kg (N = 8). ***: p < 0.001 in comparison with poor performers.

After the habituation period in the animal facility, the animals were trained in the two-way shuttle box. Along the seven daily training sessions, the animals were divided into good and poor performance groups based on the number of avoidance responses. The good performance group presented a continuous increase in the number of avoidance responses (interaction factor F_(12,1790)_ = 29.487; p = 0.000000; Fig. 2 A) and a reduction in the percentage of freezing (interaction factor F_(4,766)_ = 19.501; p = 0.00000; Fig. 2 B).

#### 3.1.2 Systemic pharmacological DA manipulation

On the 8th day, animals with good performance (N = 36) were randomly divided into five groups: 1) saline (N = 7), 2) dopamine D1 receptor antagonist SCH 0.025 mg/kg (N = 7), 3) dopamine D1 receptor antagonist SCH 0.05 mg/kg (N = 6), 4) dopamine D2 receptor antagonist sulpiride 20 mg/kg (N = 8), and 5) dopamine D2 receptor antagonist sulpiride 40 mg/kg (N = 8). After systemic drug administration, the behavior was evaluated in the two-way shuttle box and open field. Groups SCH 0.025 mg/kg and SCH 0.05 mg/kg showed a decreased number of avoidance responses after drug administration (F_(8,122)_ = 3.4368; p = 0.00133; Fig. 2 C). No changes were observed in freezing behavior (F_(8,122)_ = 0.20408; p = 0.98969; Fig. 2 D) or in general motor activity (F_(4,31)_ = 0.23501; p = 0.91648; Fig. 2 E).

#### 3.1.3 c-Fos immunohistochemistry

**Fig. 3:**
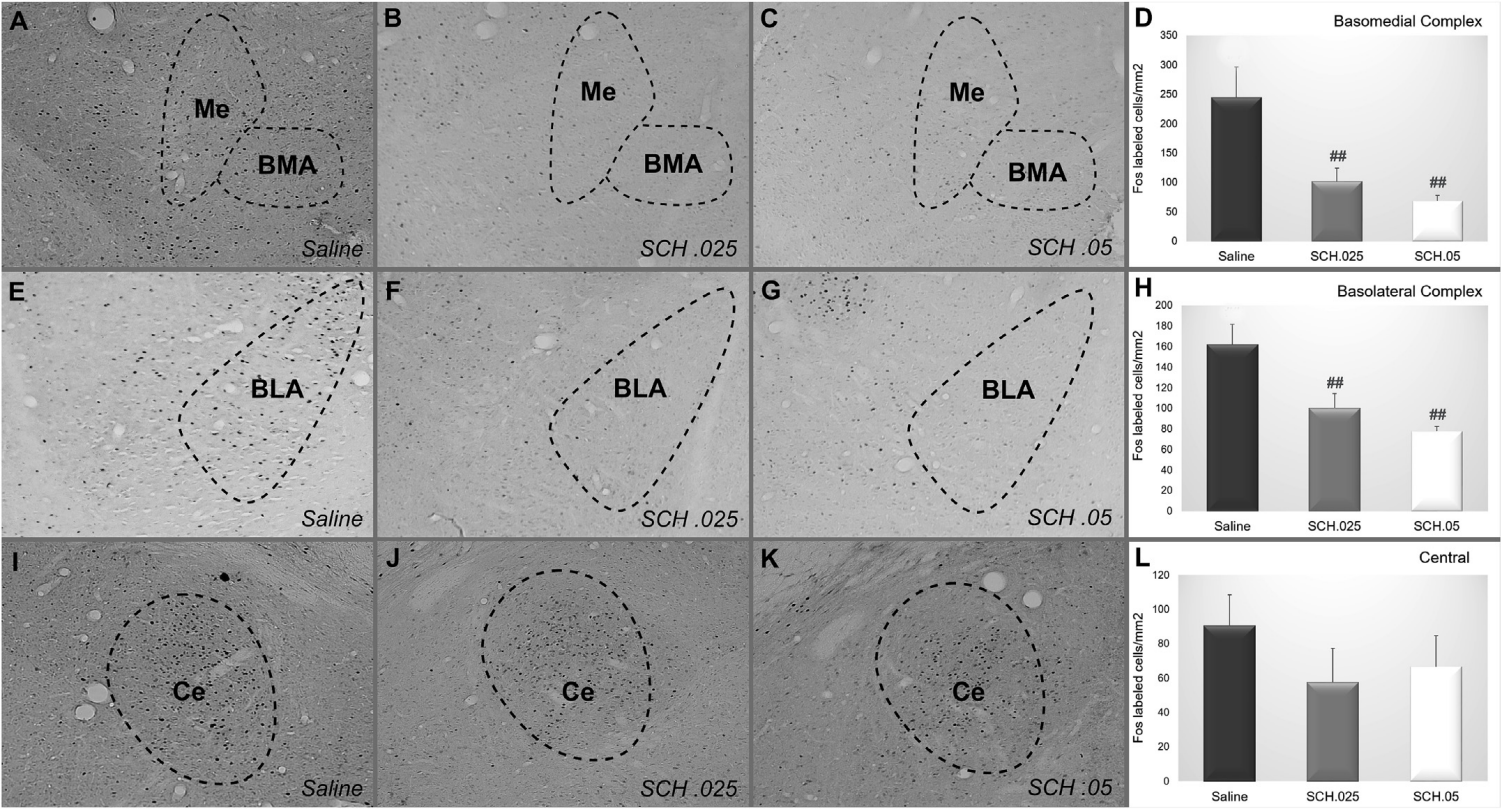
Representative photomicrographs of c-Fos staining in the basomedial complex, basolateral complex and central amygdala after the administration of (A, E, I) saline, (B, F, J) SCH 0.25 mg/kg or (C, G, K) SCH 0.5 mg/kg in good performers. Mean density of c-Fos + cells in (D) Basomedial complex, (H) Basolateral complex and (L) Central amygdala for good avoiders after the systemic injection of saline (N = 6), SCH 0.25 mg/kg (N = 6) or SCH 0.5 mg/kg (N = 5). Scale bars represent 400 μm in all photographs. **: p < 0.01 in comparison with the other groups.

The saline (N = 6), SCH 0.025 mg/kg (N = 6) and SCH 0.05 mg/kg (N = 5) groups were evaluated for c-Fos-positive cell density in the basolateral complex (basolateral + lateral, BLA), basomedial complex (basomedial + medial, BMA) and central nucleus (Ce) of the amygdala. Both SCH groups presented smaller c-Fos positive cell densities in relation to controls in the BMA (F_(2,14)_ = 7.0708; p = 0.00754; Fig. 3A–D) and BLA (F_(2,14)_ = 8.5815; p = 0.00369; Fig. 3E–H), and no differences were observed in the Ce (F_(2,14)_ = 0.82084; p = 0.46017; Fig. 3I–L). Additional data regarding c-Fos expression of other amygdala subnuclei and in SCH 0.025 mg/kg, SCH 0.05 mg/kg, sulpiride 20 mg/kg and sulpiride 40 mg/kg groups are shown in Supplementary Table 1.

#### 3.1.4 Intra-amygdala pharmacological DA manipulation

**Fig. 4:**
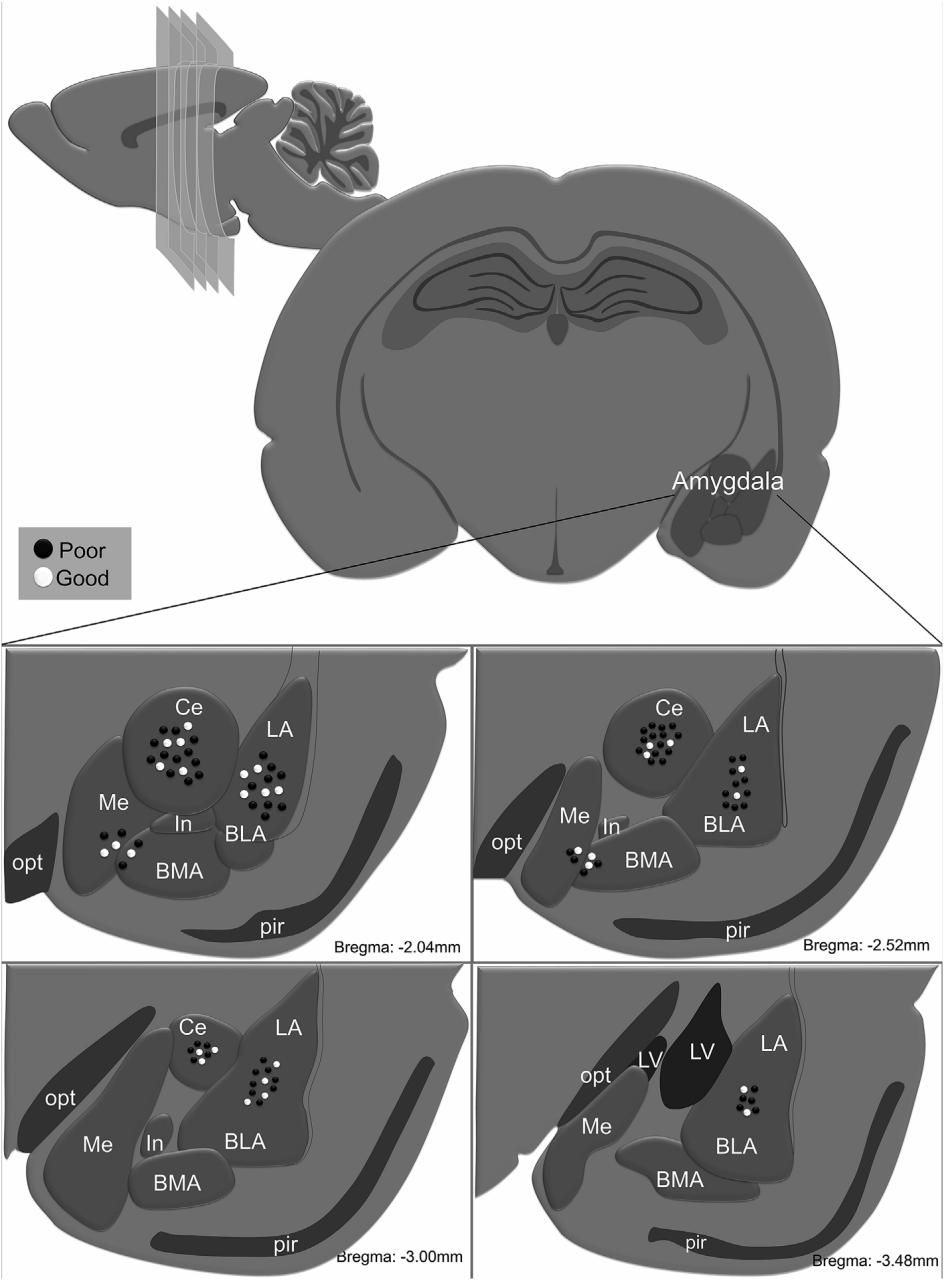
Representative microinjection sites for basomedial complex, basolateral complex and central nucleus of amygdala along bregma −2.04, −2.52, −3.00 and −3.48 mm. Each dot indicates the site of injection corresponding to poor avoiders (black dots) and good avoiders (white dots). BLA: basolateral nucleus of amygdala, BMA: basomedial nucleus of the amygdala; Ce: central nucleus of amygdala, In: intercalated nucleus of the amygdala, LA: lateral nucleus of amygdala, LV: lateral ventricle, Me: medial nucleus of amygdala; pir: piriform nucleus, opt: optic tract.

**Fig. 5:**
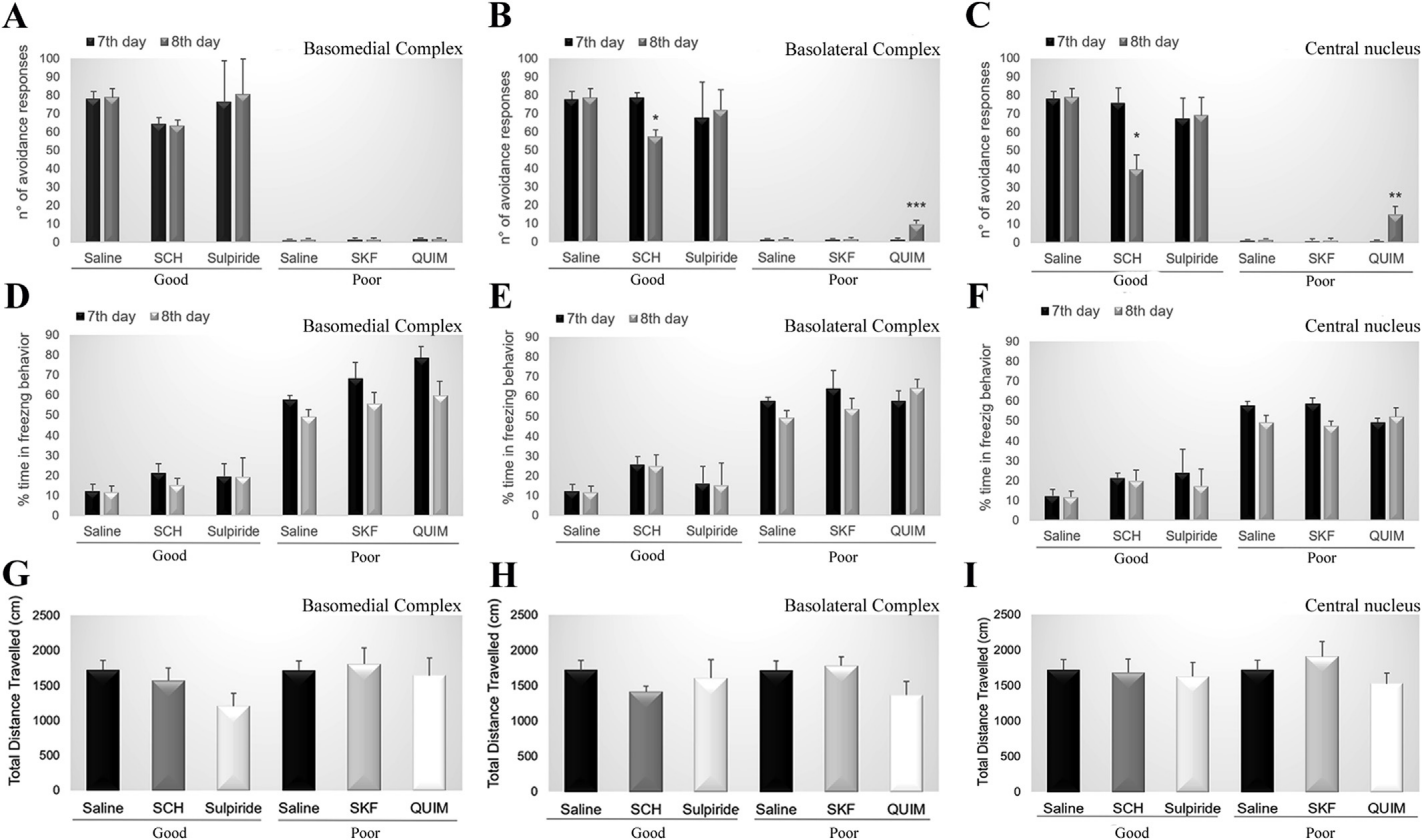
Number of avoidance performed by good performers on the 7th day of training (basal) and on the 8th day of training after the administration of saline, SCH 0.3 μL, sulpiride 0.2 μL and by poor avoiders on the 7th day of training (basal) and on the 8th day of training after the administration of saline, SKF 0.4 μL, or quinpirole 0.1 μL into the (A) basomedial complex, (B) basolateral complex or (C) central nucleus of amygdala. Percentage of time spent in freezing behavior performed by good performers on the 7th day of training (basal) and on the 8th day of training after the administration of saline, SCH 0.3 μL, sulpiride 0.2 μL and by poor avoiders on the 7th day of training (basal) and on the 8th day of training after the administration of saline, SKF 0.4 μL, or quinpirole 0.1 μL into the (D) basomedial complex, (E) basolateral complex or (F) central nucleus of the amygdala. Total travel distance showed by good performers after the administration of saline, SCH 0.3 μL, sulpiride 0.2 μL and by the poor avoiders on the 7th day of training (basal) and on the 8th day of training after the administration of saline, SKF 0.4 μL, or quinpirole 0.1 μL into the (G) basomedial complex, (H) basolateral complex or (I) central nucleus of amygdala. Group good avoiders: saline (N = 9), SCH 0.3 μL (basomedial N = 6, basolateral N = 9, central N = 9), sulpiride 0.2 μL (basomedial N = 5, basolateral N = 6, central N = 6). Group poor avoiders: saline (N = 9), SKF 0.4 μL (basomedial N = 4, basolateral N = 9, central N = 9) or quinpirole 0.1 μL (basomedial N = 4, basolateral N = 9, central N = 9). *: p < 0.05 in comparison with the corresponding 7th day.

Prior to training in the two-way shuttle box, animals were submitted to stereotaxic surgery for bilateral cannula placement in the BMA, BLA or Ce. Fig. 4 illustrates the cannula placement. On the 8th day, animals with good performance were randomly divided into groups 1) Saline (N = 9), 2) Dopamine D1 receptor antagonist SCH (BMA N = 6, BLA N = 9, Ce N = 9), and 3) Dopamine D2 receptor antagonist sulpiride (BMA N = 5, BLA N = 6, Ce N = 6) and the poor performers were randomly divided into three groups 1) Saline (N = 9), 2) Dopamine D1 receptor agonist SKF (BMA N = 4, BLA N = 9, Ce N = 9), and 3) Dopamine D2 receptor agonist quinpirole (BMA N = 4, BLA N = 9, Ce N = 9). After drug administration, behavior was evaluated in the two-way shuttle box and open field. No differences were observed in avoidance behavior in animals with cannula targeting the BMA (good: F_(2,25)_ = 0.2462; p = 0.97571, poor: F_(4,54)_ = 0.01181; p = 0.99972; Fig. 5 A). For the BLA and Ce cannula groups, SCH animals presented decreased avoidance behavior (BLA: F_(2,31)_ = 4.3462; p = 0.02168; Ce F_(2,31)_ = 3.7541; p = 0.03468; Fig. 5B and C), and quinpirole animals showed increased avoidance behavior (BLA: F_(4,94)_ = 5.3927; p = 0.00059; Ce F_(4,94)_ = 4.5334; p = 0.00216; Fig. 5B and C). No changes were observed in freezing behavior (BMA: good F_(2,25)_ = 0.35890; p = 0.70198, poor: F_(4,54)_ = 0.51430; p = 0.72548; BLA: good F_(2,31)_ = 0.00009; p = 0.99991, poor: F_(4,94)_ = 0.73201; p = 0.57235; Ce: good F_(2,31)_ = 0.00962; p = 0.99043, poor: F_(4,94)_ = 1.4678; p = 0.21821; Fig. 5D–F) or in general motor activity (BMA: F_(4,27)_ = 0.21867; p = 0.92570; BLA: F_(4,40)_ = 1.8856; p = 0.13183; Ce: F_(4,40)_ = 0.63915; p = 0.63765; Fig. 5G–I).

### 3.2 Experiment 2 – basal DA levels

#### 3.2.1 Behavior assessment

**Fig. 6:**
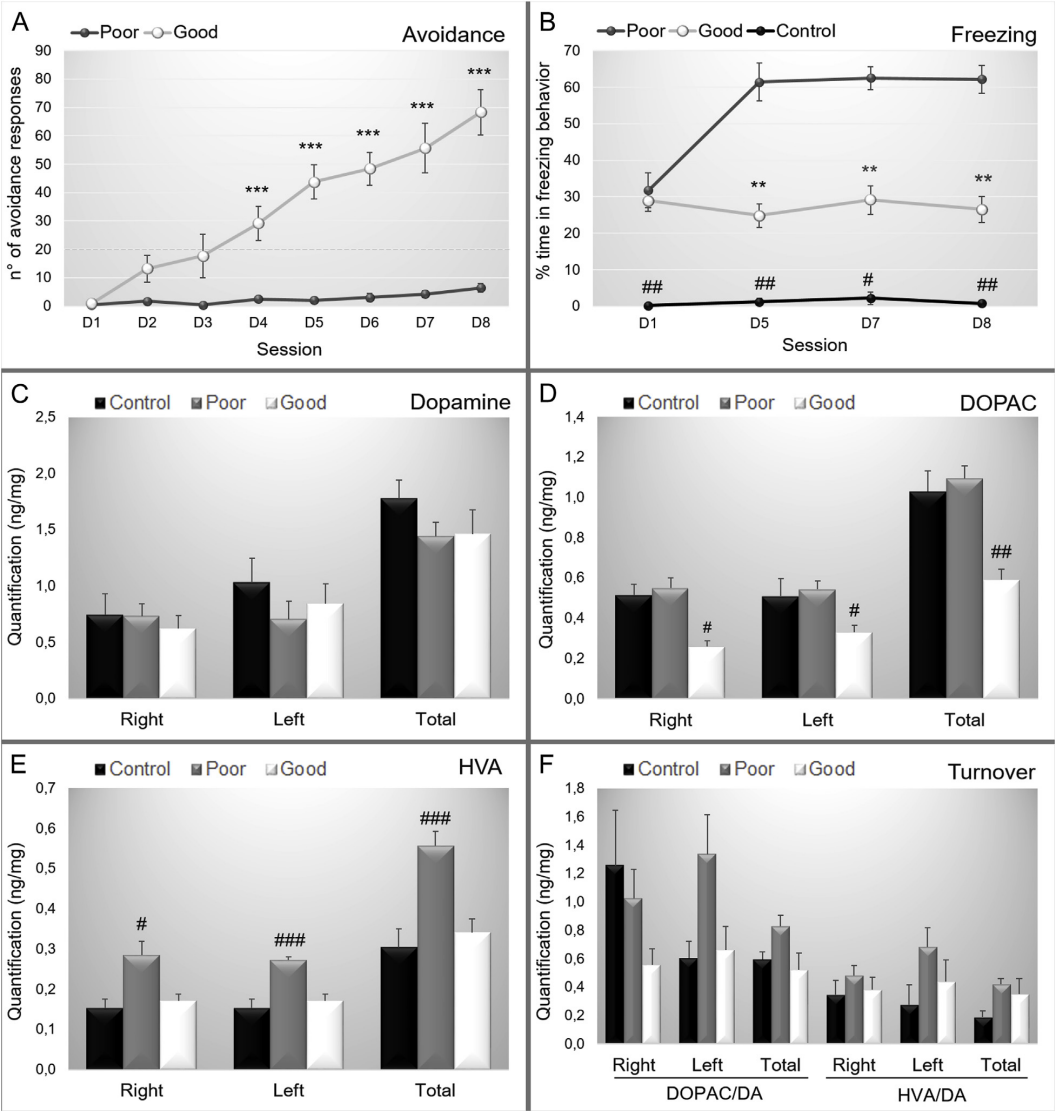
Data are reported as the means ± SEM. (A). Number of active avoidance responses in good and poor performers across training sessions. (B). Percentage of time spent in freezing behavior along sessions. (C). Quantification of dopamine (ng/mg of tissue) in the right, left and both hemispheres considering control, poor and good performers. (D). Quantification of 3,4-dihydroxyphenylacetic acid - DOPAC (ng/mg of tissue) in the right, left and both hemispheres considering control, poor and good performers. (E). Quantification of homovanillic acid - HVA (ng/mg of tissue) in the right, left and both hemispheres considering control, poor and good performers. (F). Quantification of dopamine turnover of DOPAC and HVA in the right, left and both hemispheres considering control, poor and good performers. Poor: poor performers, Good: good performers, Control: control group. DOPAC/DA: turnover rate for DOPAC; HVA/DA: turnover rate for HVA, right: right hemisphere, left: left hemisphere, total: right and left hemisphere. For the behavioral data: control (N = 5), good (N = 13) and poor (N = 15). For the HPLC data: control (N = 5), good (N = 8) and poor (N = 10) and **: p < 0.01 in comparison with poor performers, ***: p < 0.001 in comparison with poor performers, #: p < 0.05 in comparison with good and poor performers, ##: p < 0.01 in comparison with good and poor performers; ###: p < 0.001 in comparison with good and poor performers.

After a habituation period in the animal facility, the animals were randomly divided into experimental or control groups (see Fig. 1B – Experiment 2). During the training sessions, the experimental group was divided into good performance (N = 13) and poor performance (N = 15) groups based on the avoidance response presented in the two-way shuttle box. The good performance group presented a continuous increase in the number of avoidance responses (interaction factor F_(14,414)_ = 4.9798; p = 0.0000; Fig. 6 A) and a reduction in the percentage of freezing (interaction factor F_(12,288)_ = 2.3964; p = 0.00610; Fig. 1 B). Control animals were exposed to the shuttle box but received no footshock; thus, control animals presented a low percentage of freezing behavior and no avoidance responses (Fig. 6 B).

#### 3.2.2 Dopamine and metabolite quantification

The concentration of dopamine and metabolites (DOPAC and HVA) was evaluated in the total amygdala of controls (N = 5), good (N = 8) and poor (N = 10) performers. There were no differences in dopamine levels between groups in the right (F_(2,20)_ = 0.17934; p = 0.83715), left (F_(2,20)_ = 0.59812; p = 0.55938) or total amygdala (F_(2,20)_ = 0.67928; p = 0.51830; Fig. 6 C). However, the metabolite quantification showed lower levels of DOPAC in good performers (Right: F_(2,20)_ = 3.5959 p = 0.04634; Left: F_(2,20)_ = 4.4693 p = 0.02486; Total: F_(2,20)_ = 7.3101 p = 0.00414; Fig. 6 D) and higher levels of HVA in poor performers (Right: F_(2,20)_ = 5.0862 p = 0.01638; Left: F_(2,20)_ = 15.663 p = 0.00008; Total: F_(2,20)_ = 10.482 p = 0.00077; Fig. 6 E). No differences were observed in dopamine turnover in the right (DOPAC/DA: F_(2,20)_ = 1.6276; p = 0.22136; HVA/DA: F_(2,20)_ = 0.24807; p = 0.78191), left (DOPAC/DA: F_(2,20)_ = 2.4942; p = 0.10787; HVA/DA: F_(2,20)_ = 1.9352; p = 0.17049) or total amygdala (DOPAC/DA: F_(2,20)_ = 2.6346; p = 0.09647; HVA/DA: F_(2,20)_ = 1.9714; p = 0.16540; Fig. 6 F).

#### 3.2.3 Volumetric analysis

**Fig. 7:**
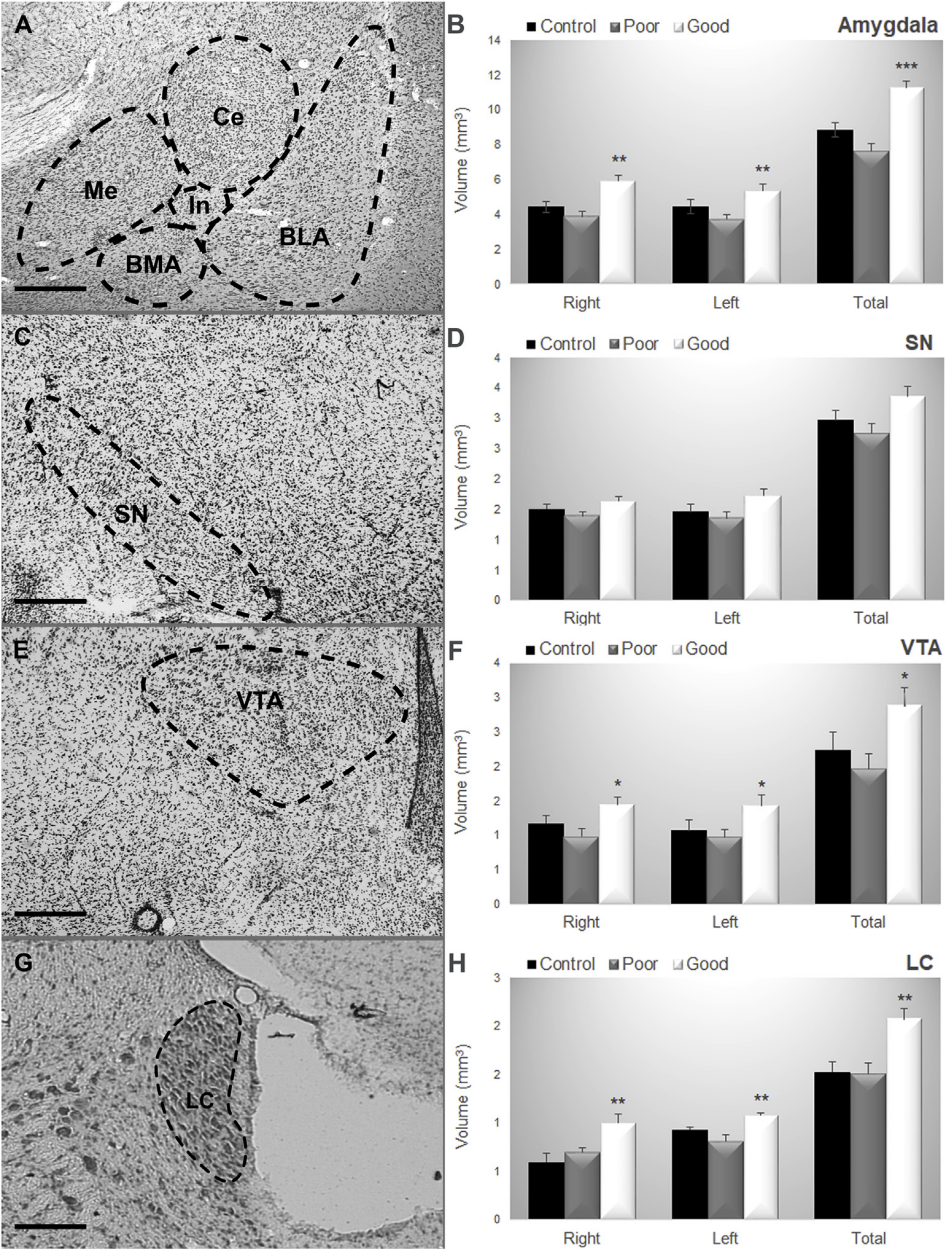
Representative photomicrographs of (A) the Amygdala nuclei, (C) Substantia Nigra, (E) Ventral Tegmental Area, (G) Locus Coeruleus. Data are reported as the means ± SEM. Volumetric estimates in (B) the Amygdala nuclei, (D) Substantia nigra, (F) Ventral Tegmental Area, (H) Locus Coeruleus considering the right and left hemisphere and the total volume considering control, poor and good performers. BLA: basolateral nucleus of amygdala; BMA: basomedial nucleus of amygdala; Ce: central nucleus of amygdala, LC: locus coeruleus; Me: medial nucleus of amygdala; SN: substantia nigra; VTA: ventral tegmental area. Control (N = 3), Good (N = 5), Poor (N = 5). Scale bars represent 400 μm in all photographs. *: p < 0.05 in comparison with poor performers; **: p < 0.01 in comparison with poor performers, ***: p < 0.001 in comparison with poor performers.

The volume of the amygdala, substantia nigra (SN), ventral tegmental area (VTA) and locus coeruleus (LC) was estimated in control (N = 3) good (N = 5) and poor (N = 5) performers. The Newman-Keuls test showed that the good performance group presented greater volume in 1) Amygdala (Right: F_(2,10)_ = 6.60; p = 0.01; Left: F_(2,10)_ = 5.96; p = 0.02, Total: F_(2,10)_ = 9.49; p = 0.005; Fig. 2, Fig. 7) VTA (Right: F_(2,10)_ = 5.00; p = 0.03; Left: F_(2,10)_ = 4.33; p = 0.04; Total: F_(2,10)_ = 4.71; p = 0.036; Fig. 3, Fig. 7) LC (Right: F_(2,10)_ = 8.38; p = 0.007; Left: F_(2,10)_ = 5.71; p = 0.02; Total: F_(2,10)_ = 9.04; p = 0.006; Fig. 7G and H). The Newman-Keuls test did not show difference in the volume of the SN (Right: F_(2,10)_ = 1.49; p = 0.27; Left: F_(2,10)_ = 2.11; p = 0.17; Total: F_(2,10)_ = 2.40; p = 0.14; Fig. 7C and D).

#### 3.2.4 TH immunohistochemistry

**Fig. 8:**
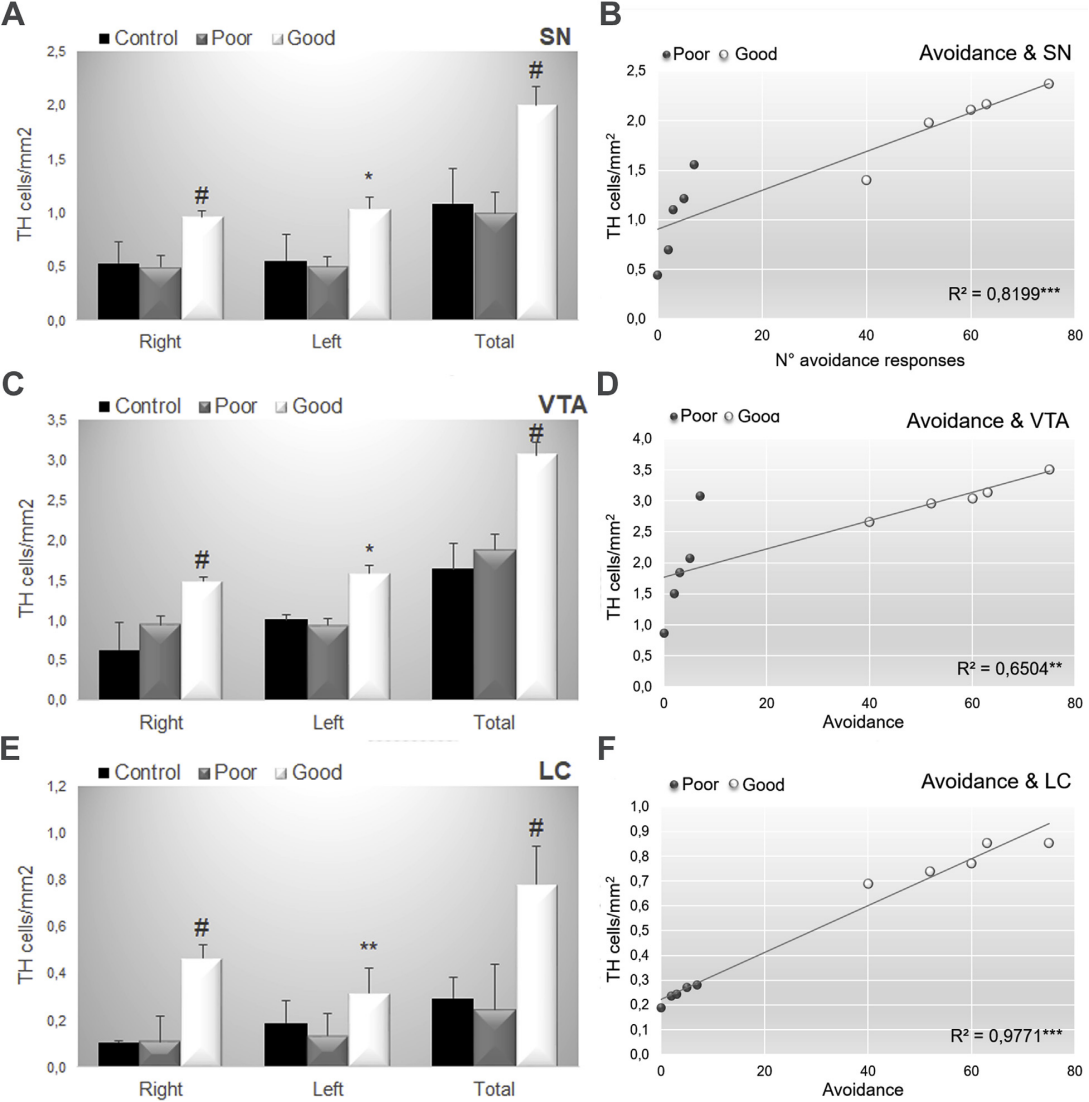
Data are reported as the means ± SEM. TH positive cells/mm2 in (A) Substantia nigra, (C) Ventral Tegmental Area, (E) Locus Coeruleus considering the right and left hemisphere and the total volume considering control, poor and good performers. Correlation data between the number of avoidance and tyrosine hydroxylase-positive cells/mm2 in the (B) Substantia Nigra, (D) Ventral Tegmental Area and (E) Locus Coeruleus considering poor and good performers. Control (N = 3), Good (N = 5), Poor (N = 5). LC: locus coeruleus; SN: substantia nigra; VTA: ventral tegmental area. Scale bars represent 400 μm in all photographs. *: p < 0.05 in comparison with poor performers; **: p < 0.01 in comparison with poor performers, ***: p < 0.001 in comparison with poor performers, #: P < 0.05 in comparison with all groups.

TH^+^ neurons were evaluated in the SN, VTA and LC of control (N = 3) good (N = 5) and poor (N = 5) performers. The Newman-Keuls test showed that the good performance group showed greater staining in all structures 1) SN (Right: F_(2,10)_ = 5.61; p = 0.02; Left: F_(2,10)_ = 5.59; p = 0.00.023; Total: F_(2,10)_ = 7.53; p = 0.011; Fig. 2, Fig. 8) VTA (Right: F_(2,10)_ = 4.59; p = 0.04; Left: F_(2,10)_ = 4.67; p = 0.00.037; Total: F_(2,10)_ = 7.97; p = 0.008; Fig. 3, Fig. 8) LC (Right: F_(2,10)_ = 138.56; p = 0.0001; Left: F_(2,10)_ = 6.77; p = 0.014 Total: F_(2,10)_ = 155.78; p = 0.0001; Fig. 8E). Furthermore, there was a significant correlation between volume and the number of avoidance responses in all structures (SN: R^2^ = 0.8199; p < 0.00031, VTA: R^2^ = 0.6504; p < 0.00482; LC: R^2^ = 0.9771; p < 0.00000; Fig. 8B, C, D).

## 4 Discussion

The present work showed that there is a distinct dopaminergic pattern for good and poor performers that is critical for the expression of avoidance behavior. Moreover, it could be possible to switch from good to poor performer, and vice versa, by intra-amygdala (BLA and Ce) injections of D1 receptor antagonist drugs in good performers or D2 receptor agonist drugs in poor performers. Furthermore, in comparison with poor performers, the good performers had lower levels of DOPAC in the amygdala, an increase in the total volume of the AMG, SN, VTA and LC, and an increase in the number of TH + cells in the SN, VTA and LC, which positively correlated with the avoidance behavior. Taken together, our results suggest that the dopaminergic system could modulate avoidance behavior.

The behavioral distinction between good and poor avoiders is well established in the literature (Choi et al., 2010; Lázaro-Muñoz et al., 2010; Martinez et al., 2013; Galatzer-Levy et al., 2014). The poor performers failed in the transition from freezing to avoidance behavior even after training (Campese et al., 2016; LeDoux et al., 2017; Boeke et al., 2017).

Considering dopaminergic modulation, our data showed that systemic injection of SCH23390, a D1 antagonist drug, could disrupt the avoidance response in good performers without affecting motor activity. These data have been supported by previous published papers that showed that subtype-dopaminergic receptors modulate avoidance (Beninger et al., 1989; Aguilar et al., 2000; Reis et al., 2004; Boschen et al., 2011; Wietzikoski et al., 2012). According to our data, the substrate for this systemic modulation is a reduction in the c-Fos activation of the basomedial and basolateral nucleus of the amygdala. A possible reason for this result could be the main localization of D1 receptors in both nuclei (Weiner et al., 1991; Boyson et al., 1986). Specifically, the anatomical pathway that regulates approach-avoidance conflict behavior could be from the medial nucleus projecting to the ventromedial hypothalamus and bed nucleus of the stria terminalis through the D1 receptor neuron population (Miller et al., 2019).

Regarding D2 receptors, the systemic injection of sulpiride, a D2 antagonist, did not affect the avoidance responses or motor activity exhibited by the good performers. Previous papers (Reis et al., 2004; Boschen et al., 2011) showed a reduction in avoidance response after systemic administration of sulpiride. However, a possible reason for these discrepant results could be the training protocol: unsignaled avoidance in our experiment versus signaled protocols (Reis et al., 2004; Boschen et al., 2011). It has been shown that unsignaled and signaled protocols have different learning pattern (Ulrich et al., 1964; Powell, 1976; Mertens et al., 2018; Hurtado-Parrado et al., 2019) and different neuroanatomical pathways (Troncoso et al., 1998; Cohen and Castro-Alamancos, 2007, 2010; Hormigo et al., 2016, 2019). For instance, in signaled protocols there is the involvement of the sensory thalamus, which fires during signaled avoidance responses (Cohen and Castro-Alamancos, 2010). Additionally, there is the projection of the substantia nigra pars reticulata, a main GABAergic output, to the superior colliculus, which is responsible for the expression of signaled avoidance responses (Hormigo et al., 2016). An interesting hypothesis concerns the possible role of dopaminergic inputs to the superior colliculus, which could contribute to the avoidance response (Essig and Felsen, 2016). Another explanation could be the possibility that the signaled protocol modulates the anxiety levels considering that there is predictability and control over the harmful outcome (Sheynin et al., 2014; Ng and Lovibond, 2017; Hormigo et al., 2019). In this line with this thinking, it has been proposed that different mechanisms could underlie the aversive response depending on the experimental protocol (Louilot et al., 1986; Campese et al., 2016, 2013).

Considering our microinjection target, the importance of basolateral and central nuclei for avoidance behavior has been reported in several papers (Werka et al., 1978; Wilensky et al., 2000; Rorick-Kehn and Steinmetz, 2005; Lázaro-Muñoz et al., 2010; Moscarello and LeDoux, 2013; Jiao et al., 2015). Specifically, the role of the central amygdala in avoidance has been investigated in active and passive avoidance protocol with different results. In passive avoidance CeA lesions impaired avoidance behavior (Grossman et al., 1975; Jellestad and Bakke, 1985). In active avoidance, Grossman et al. (1975) showed that lesions in CeA consistently produced facilitatory effects on active avoidance, which is supported by more recent studies (Lázaro-Muñoz et al., 2010; Choi et al., 2010). The reason for this apparently contradictory effect could be the technical method for performing the lesion, i.e., older studies lesions were often large and/or not particularly specific to the amygdala subnuclei; and different avoidance protocol.

The microinjection of the D1 antagonist into the basolateral and central nuclei of the amygdala decreased the number of avoidances in the good performers. The basolateral complex receives dopaminergic innervation from the ventral tegmental area (Swanson, 1982; Nader and LeDoux, 1999; de la Mora et al., 2010) and has D1 and D2 dopaminergic type receptors (Weiner et al., 1991; Scibilia et al., 1992; Levey et al., 1993; Rouillard and Freeman, 1995; de la Mora et al., 2010), while the central nucleus receives innervation from the SN (Rouillard and Freeman, 1995) and has D1 and D2 type receptors (Weiner et al., 1991; Scibilia et al., 1992). It has been shown that microinjection of D2 (sulpiride) or D1 (SCH23390) dopaminergic antagonist in the nucleus accumbens reduced the number of avoidance responses (Wadenberg et al., 1990; Wietzikoski et al., 2012). However, the administration of sulpiride into the prefrontal cortex and dorsal striatum and SCH233990 in the dorsal striatum did not affect avoidance responses (Wadenberg et al., 1990; Wietzikoski et al., 2012), suggesting that the microinjection site is the crucial factor. Supporting our data, de la Mora et al. (2010) proposed that dopaminergic modulation in the central nucleus is responsible for modulating avoidance.

The role of dopamine in acquisition is more consistent than in the expression of the avoidance behavior. It has been shown that impairment in the dopaminergic system elicits severe deficit in avoidance acquisition (Fibiger et al., 1974; Beninger, 1983; Koob et al., 1984; Wadenberg and Hicks, 1999; Darvas et al., 2011), while for avoidance expression, some studies have shown that DA is involved (Wadenberg et al., 1990; Takamatsu et al., 2015), while others have shown that it is not (Dunn et al., 1986; Nasello and Felicio, 1990). A possible reason for the discrepant results could be the variety of avoidance protocols used in the literature (Nasello and Felicio, 1990; Johnson et al., 2001; Declercq and De Houwer, 2008; Maia, 2010). Supporting this suggestion, a phasic dopamine release pattern has been shown depending on the experimental protocol (Oleson et al., 2012; Wenzel et al., 2018; Pultorak et al., 2018). Specifically, warning signal protocols release dopamine in comparison with unavoidable footshock which suppress dopamine release (Overmier and Seligman, 1967; Oleson et al., 2012), supporting the hypothesis that the protocol is an essential aspect for explaining the variety of results concerning dopaminergic modulation. Another confounder could be the dopaminergic role in encoding value for the signals. It has also been shown a phasic dopaminergic pattern depending on the value signal (Bromberg-Martin et al., 2010; Fiorillo, 2013; Gentry et al., 2016; Pultorak et al., 2018). Another possibility of bias is the testing condition. When an animal is paired with a conspecific that is shocked, the non-shocked rat exhibits an increase in the DA release, reflecting the importance of the test environment in the results (Lichtenberg et al., 2018). An acute dopaminergic reduction can also have different results on avoidance learning varying with the age of the animals that were used (Kelm & Boettiger, 2015). A last interesting possibility is that dopamine has different roles in avoidance behavior considering the concepts of safety, opponency and controllability (Huys and Dayan, 2009; Lloyd and Dayan, 2016).

A possible explanation for the differential role of D1 and D2 in poor and good performers could be due to the bidirectional modulatory role of those subtypes of receptors. The opposite modulations of D1 and D2 receptor signaling have already been shown in previous publication (Moustafa et al., 2008; Doll et al., 2011; Cox et al., 2015; Nguyen et al., 2019). There are bidirectional differences in D1 and D2 concerning transduction mechanisms. In the central nervous system, the activation of D2 receptors is through the mediation of G (βγ) proteins by inhibiting adenylate cyclase and reducing the intracellular cyclic adenosine monophosphate (cAMP) concentration while D1-receptor activation is linked to an increase in cyclic adenosine monophosphate and the activation of phospholipase C (Onali et al., 1985; Undie and Friedman, 1990; Gingrich and Caron, 1993, Jin et al., 2001; Neve et al., 2004; de la Mora et al., 2010). A bidirectional modulatory role for dopamine in avoidance through segregated D1 and D2 cortico-striatal pathways has also been proposed (Cox et al., 2014).

Dopaminergic neurons are phasically activated by alerting signals (Bromberg-Martin et al., 2010), and dysfunction in this system could contribute to an abnormal processing of environment cues in the basolateral and central nucleus of the amygdala, leading to an impairment in the avoidance response. In this sense, our data support the hypothesis (LeDoux et al., 2017) that the dopaminergic system could be considered a potential target for pharmacological therapy.

Some lines of data point to a competition between the production of the freezing response that directly opposes avoidance (Wilensky et al., 2006; Ciocchi et al., 2010; Haubensak et al., 2010; Choi et al., 2010; Lázaro-Muñoz et al., 2010). Our data regarding pharmacological intervention did not show this pattern which could be due to the variability in freezing assessments, such that in our protocol was evaluated at the beginning of the test, but in other paradigms, was evaluated throughout the session (Choi et al., 2010; Ciocchi et al., 2010). Our data is supported by the suggestion that fear levels are not crucial for affecting avoidance (Grossman et al., 1975).

Focusing on the intrinsic difference between groups, we consider that the maladaptive behavior shown by the poor performers could be attributed to the dopaminergic systems. Our data showed that good performers have a decreased DOPAC content in the amygdala without affecting DA levels and dopaminergic turnover. These data are supported by Csillag (1999), who compared brain samples of control and trained animals and showed that there is no difference in the avoidance task considering dopamine and dopamine turnover rate. Although quantitatively DA levels are the same, the mechanism of the adaptive compensatory response may be changed in these animals, specifically those related to the availability of dopaminergic receptors and the pattern of phosphorylation of the D1 and D2 receptors (Carvalho et al., 2005; Frederick et al., 2015; Yapo et al., 2017). Considering the HVA and DOPAC results, our data suggest that timing for metabolization could be responsible for the intrinsic differences between poor and good performers. To support this suggestion, Heffner et al. (1984) showed that dopaminergic metabolites are quickly eliminated during the learning process.

Other data supporting intrinsic differences could be observed in the increase in the volume and quantification of TH + cells of the SN, ventral tegmental area and locus coeruleus, as shown by the good performers that were positively correlated with the avoidance behavior. The VTA and SN contain DA neurons (Watabe-Uchida et al., 2012; Beier et al., 2015; Lerner et al., 2015; Menegas et al., 2015). The reduction in the pattern of TH neurons in the SN can lead to impairments in memory and, learning (Moreira et al., 2012) and in avoidance tasks (Díaz-Véliz et al., 2002). Additionally, monitoring the substantia nigra and VTA volume could be an index of cognitive status, considering that their volume has been considered a parameter of the progression of neurodegenerative diseases (Chen et al., 2014; De Marco and Venneri, 2018; D'Amelio et al., 2018). Additionally, the dopaminergic system controls motivation, and electrical stimulation of the VTA has been suggested as an effective target for depressive disorders (Yadid and Friedman, 2008; Friedman et al., 2009; 2012; Gazit et al., 2015).

The LC is the main noradrenergic nucleus (Schwarz and Luo, 2015; Feinstein et al., 2016) that has been suggested to be responsible for the elaboration of the avoidance response (Shelkar et al., 2016; Clewett et al., 2018) probably due to the locus coeruleus-amygdala circuitry (Sabban et al., 2018). Clarifying the elaboration of the avoidance, intra-LC injections of the putative neurotransmitter agmatine increases the inhibitory avoidance mediated by alfa2-adrenoreceptors and nitric oxide (Shelkar et al., 2016). Further, approximately a month after the inhibitory avoidance test, there is an increase in noradrenergic receptors, neuropeptide Y receptors, corticotropin-releasing hormone receptors and the endocannabinoid system in the LC and amygdala (Clewett et al., 2018). Good performers present a higher number of TH neurons than poor performers in the LC, which could be supported by a study that showed that injury of the noradrenergic system impairs instrumental avoidance probably through the loss of cells in the LC (Radwanska et al., 2010). Another possible interpretation could be that TH + cells in the LC may reflect signaling of a different catecholamine instead of only noradrenergic regulation. Supporting this hypothesis, there is close interaction between the dopaminergic and noradrenergic systems (Antelman and Caggiula, 1977; Lisieski et al., 2019; Zerbi et al., 2019), as LC stimulation induces a concomitant release of DA and NA (Devoto et al., 2001; 2003a, 2003b, 2004, 2005) and there is an anatomically specific connectomic fingerprint of LC with dopamine receptors (Zerbi et al., 2019).

In the same line of thinking, previous works from other paradigms such as reversal learning, lever press, the decision-making task and, instrumental learning support the assumption that the dopamine system is crucial for modulating individual differences (Tomie et al., 2000; Cheng and Feenstra, 2006; Randall et al., 2012; Vollbrecht et al., 2015; Klanker et al., 2015; Groman et al., 2019).

Although volumetric changes in the LC could not be directly correlated with function (Mounton et al., 1984), it could represent a cognitive status (Theofilas et al., 2017) because there is a decrease in LC volume in neurodegenerative diseases (Chen et al., 2014; Theofilas et al., 2017).

The amygdala is involved in the modulation of avoidance responses (LeDoux, 2000; Schlund and Cataldo, 2010; Lázaro-Muñoz et al., 2010; Martinez et al., 2013; Ramirez et al., 2015; Ardeshiri et al., 2017; Korn et al., 2017). Our data are in contrast with a previous published paper (Rio-Álamos et al., 2017) that showed that low avoider rats had greater amygdala volume in comparison with high avoider rats. A possible reason for this difference could be the use of high- and low-avoidance animals inbred strains, which were originally selected and bred in accordance with their performance in the avoidance task, as previously suggested by our research group (de Oliveira et al., 2016). In this same line of thinking, Mozhui et al. (2007) quantified the basolateral amygdala across 35 inbred lines, showing huge variability in volume and cell populations between strains. Additionally, Sultana et al. (2019) showed that inbred strains exhibited contrasting characteristic behaviors and differences in genetic background.

Taken together, our data have provided evidence for a dopaminergic signature of avoidance performers, emphasizing the role of distinct dopaminergic receptors in individual differences in avoidance behavior based on pharmacological, immunohistochemical, neurochemical and volumetric analyses.

Regarding technical limitations, the invasiveness of the guide cannula and the microinjection procedure, and the impossibility to perform a drug-free test on the day 9 should be considered. Additionally, the pattern of drug spread was not evaluated and there were no offsite controls; however, the drug and dose used was based on previous papers that targeted the same structure. Further, we did not infuse the agonist in the good performers or the antagonist in the poor performers as counterproof and to minimize the drug effects.

## 5 Conclusion

The results of our study provide a better understanding of the role of the dopaminergic system in the execution of avoidance behavior. Specifically, good performers have DA patterns in the amygdala, SN, VTA and LC that are intrinsically different from those of poor performers, and it could be possible to convert animals from good to poor performers through manipulation of D1 and D2 receptors. In the future, we expect that our results will provide insights into the treatment of psychiatric disorders.

## Authors’ contributions

GFA, FVA, and FSR: performed the experiments, data curation, and formal analyses, and reviewed and edited the paper; MDJS, MCC, CCO, LCTS, MCC, and MAK: performed the experiments and formal analyses, and edited the paper; MJT, JPO, MB, and ETF: conceptualized the experiments and reviewed and edited the paper; RCRM: conceptualized the experiments, obtained the funding, administered and supervised the project, and wrote the paper.

## CRediT authorship contribution statement

**Geiza Fernanda Antunes:** Formal analysis, Data curation, Writing - review & editing. **Flavia Venetucci Gouveia:** Formal analysis, Data curation, Writing - review & editing. **Fabiana Strambio Rezende:** Formal analysis, Data curation, Writing - review & editing. **Midiã Dias de Jesus Seno:** Formal analysis, Writing - review & editing. **Milene Cristina de Carvalho:** Formal analysis, Writing - review & editing. **Caroline Cruz de Oliveira:** Formal analysis, Writing - review & editing. **Lennon Cardoso Tosati dos Santos:** Formal analysis, Writing - review & editing. **Marina Correia de Castro:** Formal analysis, Writing - review & editing. **Mayra Akemi Kuroki:** Formal analysis, Writing - review & editing. **Manoel Jacobsen Teixeira:** Writing - review & editing, Conceptualization. **José Pinhata Otoch:** Writing - review & editing, Conceptualization. **Marcus Lira Brandao:** Writing - review & editing, Conceptualization, Writing - review & editing, Conceptualization. **Erich Talamoni Fonoff:** Conceptualization, Supervision, Validation, Writing - review & editing.

## Declaration of competing interest

The authors report no conflicts of interest.
