# Supplementary material for: Dopamine modulates individual differences in avoidance behavior: A pharmacological, immunohistochemical, neurochemical and volumetric investigation
Source: Neurobiol Stress. 2020 Apr 8;12:100219. doi: 10.1016/j.ynstr.2020.100219 (PMC7231994; doi:10.1016/j.ynstr.2020.100219)
Supplement: Multimedia component 1 [file mmc1.docx]

| Supplementary Table1. c-Fos positive cells density. | | | | | | |
| --- | --- | --- | --- | --- | --- | --- |
| **Nucleus** | **Saline** | **SCH.025** | **SCH.05** | **Sul20** | **Sul40** | **ANOVA** |
| Cel | 25.14 ± 5.74 | 18.72 ± 8.77 | 24.38 ± 14.87 | 16.13 ± 5.81 | 48.55 ± 11.98 | F_(4,21)_= 1,88 p= 0,150 |
| Cem | 28.99 ± 7.31 | 29.41 ± 18.68 | 19.62 ± 6.49 | 22.96 ± 8.69 | 34.05 ± 11.10 | F_(4,17)_= 0,27 p= 0,892 |
| Cec | 31.33 ± 9.47 | 21.05 ± 9.81 | 18.97 ± 8.3 | 23.41 ± 13.06 | 57.21 ± 14.26 | F_(4,21)_= 1,78 p= 0,169 |
| CeTotal | 28.48 ± 5.66 | 21.82 ± 8.39 | 20.99 ± 9.96 | 20.83 ± 8.19 | 48.54 ± 11.82 | F_(4,21)_= 1,65 p= 0,200 |
| LAd | 16.49 ± 2.60 | 9.91 ± 3.27 | 24.16 ± 12.91 | 14.96 ± 3.85 | 30.18 ± 7.50 | F_(4,21)_= 1,73 p= 0,181 |
| LAvm | 20.61 ± 6.43 | 21.64 ± 3.78 | 9.28 ± 1.22 | 24.67 ± 14.21 | 30.00 ± 5.21 | F_(4,16)_= 0,83 p= 0,521 |
| LAvl | 13.67 ± 2.22 | 14.59 ± 2.62 | 10.54 ± 1.11 | 20.78 ± 7.49 | 41.26 ± 11.00 | F_(4,16)_= 2,97 p= 0,051 |
| LATotal | 16.93 ± 3.00 | 14.61 ± 0.86 | 23.55 ± 13.20 | 17.53 ± 5.66 | 29.12 ± 6.11 | F_(4,21)_= 1,24 p= 0,324 |
| BLAa | 29.52 ± 9.14 | 20.55 ± 5.38 | 12.50 ± 3.03 | 25.98 ± 11.18 | 41.91 ± 9.35 | F_(4,21)_= 1,40 p= 0,267 |
| BLAv | 32.32 ± 7.64 | 23.68 ± 3.64 | 12.74 ± 2.52 | 23.95 ± 8.53 | 26.59 ± 4.60 | F_(4,21)_= 1,02 p= 0,417 |
| BLAp | 29.90 ± 11.09 | 20.85 ± 8.30 | 11.32 ± 3.04 | 47.35 ± 3.83 | 39.94 ± 10.00 | F_(4,16)_= 1,29 p= 0,314 |
| BMAa | 17.27 ± 7.72 | 34.84 ± 11.62 | 14.92 ± 6.44 | 31.76 ± 10.87 | 33.52 ± 7.96 | F_(4,17)_= 1,05 p= 0,409 |
| BMAp | 24.01 ± 7.35 | 50.05 ± 22.25 | 3.93 ± 1.49 | 48.54 ± 8.14 | 34.70 ± 9.30 | F_(4,16)_= 1,54 p= 0,237 |
| BLATotal | 26.60 ± 5.63 | 28.60 ± 5.54 | **12.90 ± 4.21*** | 31.04 ± 9.82 | 33.58 ± 6.10 | F_(4,18)_= 5,71 p= 0,003 |
| MEad | 191.21 ± 67.55 | 12.56 ± 1.31 | 124.04 ± 104.82 | 60.52 ± 16.21 | 117.99 ± 43.26 | F_(4,15)_= 1,01 p= 0,432 |
| MEav | 157.87 ± 85.14 | 30.42 ± 0,85 | 114.70 ± 114.70 | 92.17 ± 34,69 | 182.77 ± 88.89 | F_(4,15)_= 0,33 p= 0,852 |
| MEpd | 133.76 ± 55.23 | 13.21 ± 1.43 | 16.74 ± 4.91 | 143.36 ± 71.06 | 100.75 ± 33.48 | F_(4,20)_= 2,39 p= 0,084 |
| MEpv | 119.87 ± 54.67 | 13.10 ± 3.14 | 17.30 ± 0.23 | 47.32 ±10.56 | 90.96 ± 35.20 | F_(4,20)_= 1,87 p= 0,153 |
| METotal | **150.68 ± 60.94^#^** | 13.71 ± 2.22 | 80.80 ± 64.54 | 79.15 ± 25.42 | **133.07 ± 47.77^#^** | F_(4,20)_= 3,67 p= 0,021 |
| PrL | 27.99 ± 9.70 | 42.69 ± 8.93 | 16.53 ± 10.82 | 25.21 ± 10.82 | 26.22 ± 9.03 | F_(4,20)_= 0,87 p= 0,495 |
| IL | 28.96 ± 8.72 | 21.51 ± 5.26 | 50.27 ± 43.23 | 20.15 ± 3.68 | 24.86 ± 8.58 | F_(4,20)_= 0,89 p= 0,486 |
| PFCTotal | 28.48 ± 5.79 | 32.10 ± 6.01 | 33.40 ± 26,36 | 22.68 ± 6.92 | 25.54 ± 8.72 | F_(4,20)_= 0,26 p= 0,898 |
| Abbreviations: Central Amygdala Nucleus (Ce) lateral (Cel), medial (Cem) and central (Cec) region; Lateral Amygdala Nucleus (LA) dorsal (LAd), ventromedial (LAvm) and ventrolateral (LAvl) region; Basolateral Amygdala Complex (BLA) anterior (BLAa), ventral (BLAv) and posterior (BLAp) region; Basomedial Amygdala Nucleus (BMA) anterior (BMAa) and posterior (BMAp) region; Medial Amygdala Nucleus (ME) anterodorsal (MEad), anteroventral (MEav), posterodorsal (MEpd) and posteroventral (MEpv) region; Prelimbic (PrL) and Infralimbic (IL) Prefrontal Cortex (PFC), | | | | | | |
